# Value Associations Modulate Visual Attention and Response Selection

**DOI:** 10.3389/fpsyg.2021.656185

**Published:** 2021-05-21

**Authors:** Annabelle Walle, Ronald Hübner, Michel D. Druey

**Affiliations:** Department of Psychology, University of Konstanz, Konstanz, Germany

**Keywords:** attention, monetary reward, monetary loss, Simon task, response selection, associative learning

## Abstract

Every day, we are confronted with a vast amount of information that all competes for our attention. Some of this information might be associated with rewards (e.g., gambling) or losses (e.g., insurances). To what extent such information, even if irrelevant for our current task, not only attracts attention but also affects our actions is still a topic under examination. To address this issue, we applied a new experimental paradigm that combines visual search and a spatial compatibility task. Although colored stimuli did not modulate the spatial compatibility effect more than gray stimuli, we found clear evidence that reward and loss associations attenuated this effect, presumably by affecting attention and response selection. Moreover, there are hints that differences in these associations are also reflected in a modulation of the spatial compatibility effect. We discuss theoretical implications of our results with respect to the influences of color, reward, and loss association on selective attention and response selection.

## Introduction

Many tasks of working people nowadays take place on the Internet. Although task-irrelevant, there is a constant flow of advertising, which sometimes promises rewards (the purchase of a gadget we always wanted) or plays with our fears (insurances). However, paying attention or reacting to these advertisements can be harmful to the current task. To perform a task efficiently, we have to select task-relevant information from the environment, while ignoring irrelevant information (Allport, 1989; Hübner et al., 2010), and respond correspondingly. This raises the question: Which stimulus attracts our attention to what extent? For a long time, the consensus has been that stimuli exclusively attract attention due to their specific low-level features (i.e., perceptual salience), or their consistency with the observer’s objective(s) (Theeuwes et al., 2010). This assumption has recently been challenged by reports of stimuli attracting attention due to their selection or reward history (cf. Awh et al., 2012). This means a stimulus may attract attention either because participants reacted to it in the past (e.g., Sha and Jiang, 2016) or because a reaction to it was previously paired with monetary reward or loss (e.g., Wang et al., 2013; for reviews, see Chelazzi et al., 2013; Anderson, 2016, 2019; Bourgeois et al., 2016; Failing and Theeuwes, 2018; Watson et al., 2019).

In this study, we focus on how value associations (association of a stimulus with reward or loss) modulate attentional guidance and subsequent response selection. For this objective, we examine with a new variant of the Simon task (Simon and Rudell, 1967; Hommel, 2011) how value associations modulate the spatial compatibility effect. In the following, we will provide the theoretical background for our study.

### The Guidance of Visual Attention

With regard to the question of how attention is guided in general, several theories and models of visual attention (e.g., Wolfe, 1994; Itti et al., 1998; Itti and Koch, 2000) suggest that visual information is first separated preattentively and represented on so-called feature maps. Each feature attribute of an item is contrasted with the corresponding feature attributes of the surrounding items. The resulting difference signals on each map are accumulated on a common topographically arranged map. Attention is assumed to proceed serially, guided by the strength of the activations on this map. It has been proposed that bottom-up (e.g., salience) and top-down (e.g., goals) influences as well as the selection history (e.g., reward history) of the stimuli contribute to the common activity signals (Awh et al., 2012; Failing and Theeuwes, 2018). Recently, especially reward history has inspired research (c.f. Failing and Theeuwes, 2018; Anderson, 2019).

### Value Associations and the Role of Task Relevance

The most investigated phenomenon of reward history is probably value-driven attentional capture (VDAC) (Anderson et al., 2011b; cf. Anderson, 2016). In VDAC studies (e.g., Anderson et al., 2012; Wang et al., 2013), specific target stimuli or stimulus features are associated with rewards or losses during a training phase. The training phase is followed by a test phase in which the former value-associated stimuli/features are presented as distractors, but their occurrence does not result in reward or loss. Typically, VDAC is observed in the test phase. That is, value-associated stimuli attract attention more than stimuli associated with lower or no reward (e.g., Anderson and Halpern, 2017, Experiment 1; Marchner and Preuschhof, 2018). As the correct response to the value-associated target was rewarded in the training, it was argued that attentional orientation to this target was learned (cf. law of effect, Thorndike, 1911). This learned orientation response might persist, resulting in the occurrence of VDAC in the test phase (cf. Failing et al., 2015; Le Pelley et al., 2015; Failing and Theeuwes, 2017).

However, value associations also affect attention, if the values have been exclusively associated with specific task-irrelevant distractors (see Le Pelley et al., 2016; Failing and Theeuwes, 2018; for reviews). Although in corresponding studies, the distractor signals the possibility to earn a reward, actually paying attention to the distractor usually results in losing or not obtaining money and is thus against participants’ goals (e.g., Failing et al., 2015; Le Pelley et al., 2015; Pearson et al., 2015; Failing and Theeuwes, 2017). A special feature of these studies is that the value association is often established and tested over the course of the whole experiment (but see, e.g., Mine and Saiki, 2018, Experiment 1, for a training-test approach). For instance, in the study of Le Pelley et al. (2015), a specific version of the so-called *additional-singleton task* (Theeuwes, 1992, 2010) was used. Participants searched for a shape singleton (a stimulus unique in shape) among distractor circles and categorized the orientation of a line within that stimulus. On some trials, one of the distractors was a salient color singleton whose color signaled either a high or low reward for a correct response. As a result, responses were generally slower in the presence of a high reward distractor singleton compared to one associated with low reward. By using deadline and eye-tracking procedures, the authors showed that participants allocated attention to the value-associated color singleton, even if this behavior resulted in getting less or no reward.

As described previously, in this kind of task, the associated value is gained or lost during the whole course of the experiment, and participants are often informed explicitly about the corresponding stimulus–value associations (e.g., Failing et al., 2015; Failing and Theeuwes, 2017). Thus, one might assume that the observed effects can be explained solely in terms of *incentive motivation* (cf. Chelazzi et al., 2013). Participants know whether they could win or lose money in the current trial due to the distractor color and could adapt their behavior in a way that they are able to maximize their earnings. From this point of view, however, it is unclear why the participants show a maladaptive behavior (paying attention to the distractor) instead of using a strategy that might maximize their earnings (suppressing the distractor). Failing and Theeuwes (2017, Experiment 4) argued in this context that effects driven exclusively by the participants’ explicit knowledge about the stimulus–value associations should already be observable in the beginning of corresponding experiments. They examined this assumption and were able to show that the value effects emerged gradually over time, which suggests that a learning mechanism might be the driving force of the effect.

### Reward- and Loss-Driven Effects on Attention

Although value-driven effects on attention have mostly been investigated exclusively with reward associations, in a few studies loss-associated stimuli have also been used (see Watson et al., 2019; for a review). However, investigating effects driven by reward or loss associations, where the total earnings are performance-contingent, is rather difficult. One problem is that – even if numerically equal – rewards and losses might not be perceived as equivalent in their magnitude, with losses apparently subjectively weighing more (e.g., Kahneman and Tversky, 1979; but see Yechiam and Hochman, 2013). Other problems concern the task design. Mostly, participants have to respond correctly and before a deadline to earn a reward or to avoid losing an already earned reward (e.g., Müller et al., 2016). This procedure typically results in an imbalance in learning the corresponding value associations, as the positive effect of the reward-associated stimulus is experienced more often than the negative effect of the loss-associated one (cf. Choi and Cho, 2020). An attempt has been made to work around this problem by letting participants earn less money, if the response was wrong in the reward condition, or lose less money, if the response was correct in the loss condition (e.g., Carsten et al., 2019; see also Wang et al., 2013). However, with this technique there is still an imbalance. The higher reward and the lower loss are still experienced more often than the lower reward and the higher loss. It was also proposed to use a deadline procedure, which adjusts participants’ performance in a way that only in 50% of cases a preferable feedback is shown to create a balance between reward and loss feedback (Carsten et al., 2019). However, as participants’ goal is to earn money, which is nearly impossible under these circumstances, the procedure might introduce a negative overall context frame, presumably confounding interpretations with regard to reward and loss associations.

Given these difficulties, is there nevertheless evidence that rewards and losses associated with task-irrelevant stimuli drive attention similarly? In studies, where the value association could also be integrated within a task-relevant element at any point in the experiment (e.g., since the association was learned within a target in the training), the results are inconclusive: While in some studies similar effects for task-irrelevant reward and loss associations were found (Wang et al., 2013, Experiment 1; Wentura et al., 2014), in others differences were observed highlighting a stronger influence of reward associations on attention (Gupta et al., 2016; Müller et al., 2016; Barbaro et al., 2017; see Carsten et al., 2019, supplemental material; Sun et al., 2017, for contrary results). In the study of Barbaro et al. (2017), for instance, reward-associated distractors produced stronger interference than loss-associated ones, when participants looked for a neutral target. If reward and loss were associated exclusively with specific distractors, to our knowledge, differences were only investigated in the temporal attention domain: If stimuli are presented sequentially, performance is similarly impaired, if the target follows shortly after a reward- or loss-associated distractor (Le Pelley et al., 2019).

In summary, although results are not completely conclusive, there are indications that reward associations have a stronger effect on attention than loss associations (e.g., Barbaro et al., 2017). After this short review of the literature about the influence of value associations on attention, we now consider the Simon task and response selection.

### The Simon Effect and the Theory of Event Coding

In the Simon task (Simon and Rudell, 1967; Hommel, 2011), a laterally presented stimulus has to be categorized according to, for instance, its color (e.g., whether it is red or green), by pushing a corresponding left or right button. Even though stimulus location is completely task-irrelevant, performance is usually worse for an incongruent stimulus, i.e., when the required response is opposite to the stimulus location, than for a congruent one, i.e., when the location of the required response corresponds to the stimulus location. Interestingly, there is first evidence that this spatial compatibility or *Simon* effect can also be influenced by value associations. In the study of Wang et al. (2019), the Simon effect was modulated by the reward association of the target. A low-reward association resulted in a smaller Simon effect than a high-reward association.

Different explanations have been offered so far for the Simon effect. We focus on the approach suggested within the Theory of Event Coding (TEC) (cf. Hommel et al., 2001; Hommel, 2011, 2019). According to TEC, the perceptual representation of a stimulus and its corresponding response representation are coded in the same format, based on so-called *feature codes.* Assuming that a red stimulus is presented on the left side of the screen, and the correct response key is also located on the left side of the keyboard (congruent condition). The representation of the stimulus consists of its stimulus feature codes for its color (red) and its spatial location (left). The corresponding response representation includes, for instance, the spatial position of the correct response key (left). As both representations include the same spatial feature code (left), and therefore overlap, the presentation of the stimulus also results in an automatic activation of the corresponding response representation (i.e., stimulus and response representation are bound in a common event file). If, however, the red stimulus is presented on the right, but the correct response key is on the left (incongruent condition), the activation of the stimulus feature codes (red, right) also primes the representation of the wrong response located on the right, but simultaneously, the representation for the left correct response is activated in order to react. As both response representations are activated, a response conflict emerges, which results in decreased performance, explaining the Simon effect.

### The Current Study

**FIGURE 1:**
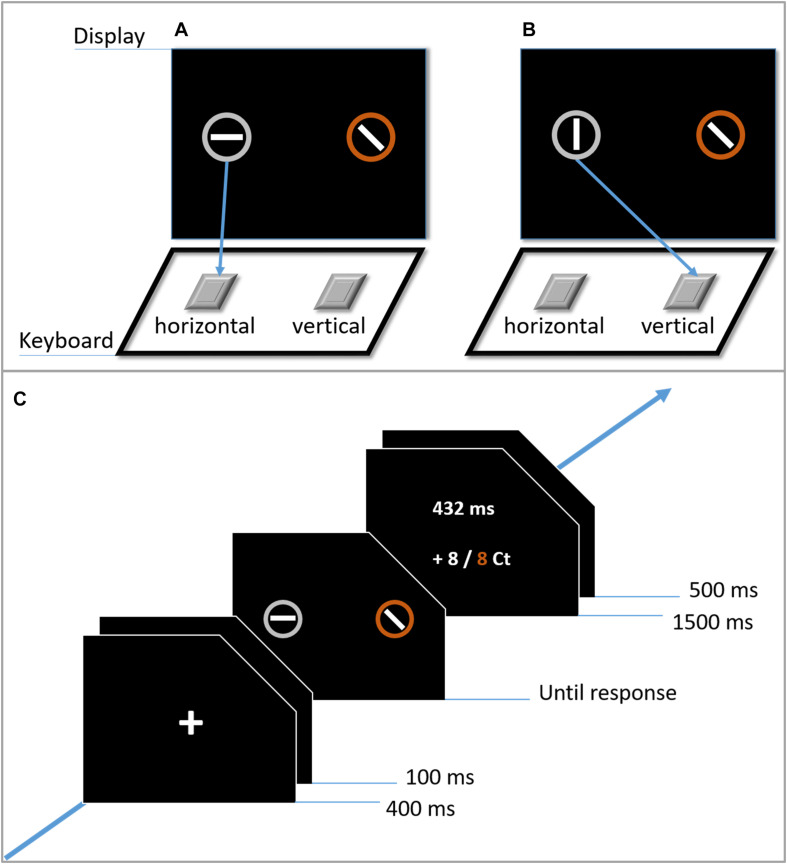
Illustration of the congruent **(A)** and incongruent **(B)** condition in the Simon search task, as well as an example trial sequence in Experiment 1 **(C)**.

In this study, we examined, how task-irrelevant (in terms of being part of a distractor) value associations modulate attention and subsequent response selection. As in other studies, circles enclosing a line were used as stimuli (e.g., Le Pelley et al., 2015). The task was to identify the target and to decide whether its line is vertical or horizontal. Different from previous studies, we presented only a single distractor. The target was shown either on the left or on the right side of the screen, whereas the distractor appeared on the opposite side. Because of the horizontal arrangement of both items (Figure 1), and given reports of Simon effects in similar task configurations (cf. Hommel, 1993), we expected a spatial compatibility effect similar to that in the Simon task (Simon and Rudell, 1967; Hommel, 2011) for the target location.

Our task can be seen as a hybrid between visual search and the Simon task. Given our task and theoretical outlines so far, what were our expectations? If target and distractor are presented on opposite sides of the display, two corresponding spatial feature codes are activated (i.e., left and right). If the distractor is salient, or even associated with reward or loss, the activity signal for this location on the common activity map should rise (cf. Failing and Theeuwes, 2018), resulting in this distractor capturing attention with a higher probability than a neutral one. Moreover, as attention is required for binding different features correctly into a common percept (Treisman and Gelade, 1980; Treisman, 1998) and, as suggested in TEC, the perceptual representation of a stimulus and its response representation overlap in the case of lateralized stimuli and response keys (cf. Hommel et al., 2001), attending the distractor should result in binding together the stimulus and response feature codes that relate to the distractor location. This in turn should increase the activation of the spatial response code and, consequently, lead to a priming of the corresponding response. This response activation is directly opposite to the response activation of the target.

The activation of the distractor response should modulate the Simon effect related to the target location: If target and response are congruent, attending the (incongruent) distractor should result in performance costs, as the incongruent response is primed. If target and response are incongruent, performance benefits are expected because of the response priming by the (congruent) distractor. This priming should reduce the response conflict caused by the target. We will use the terms congruent and incongruent only for the target-location response relationship. Thus, if the distractor is attended, as it is salient or value-associated, the described mechanisms should result in an attenuated Simon effect, compared to a case, where a less attended distractor is present. First evidence supporting these assumptions has already been provided by Proctor and Lu (1994), who showed that the Simon effect is reduced in a target–distractor display, if distractor and target are colored differently. We will elaborate the specific value-associated hypotheses in Experiment 1.

## Experiment 1

In our first experiment, we examined the extent to which value-associated stimuli modulate attention and subsequent response selection. More specifically, we examined how much value-associated distractors reduce the Simon effect. We considered four distractor types: First, a neutral one, which had the same color (gray) as the target; second, a colored baseline distractor associated with neither reward nor loss; third, a reward-associated colored distractor; and, fourth, a loss-associated colored distractor. The respective conditions are labeled *gray*, *no value*, *reward*, and *loss*. We assumed that colored distractors (i.e., no value distractors) reduce the Simon effect relative to gray ones. Moreover, the presentation of reward and loss distractors should result in a further reduction (or even a reversal) relative to no value distractors. Further, based on previous results (e.g., Barbaro et al., 2017), a reward-associated distractor should attenuate the Simon effect more than a loss-associated one.

We used reward- and loss-associated distractors, despite the mentioned difficulties in attributing corresponding effects to the particular value association. Our rationale was that finding a difference between both conditions shows that differences in the value association can modulate response selection distinctively irrespective whether this difference is due to the specific value or imbalances in the frequency of occurrence of the rewards and losses. Moreover, it is possible to examine whether reward and loss associations affect attention and response selection in a qualitatively similar way.

In line with other studies (e.g., Failing et al., 2015; Failing and Theeuwes, 2017) in which value associations were manipulated within a distractor, participants were explicitly informed about the specific color–value associations and that the distractors were task-irrelevant. With this procedure, there is no need to explore the distractors in order to figure out whether specific distractor colors might signal the possibility to earn or lose a specific monetary value. Moreover, in combination with the implementation of a deadline, participants should not be motivated to attend to the corresponding distractors, as this might result in not winning the reward or losing money with a higher probability. In summary, the explicit instruction and the deadline should ensure that influences of the different distractors could not be traced back to motivational aspects.

### Materials and Methods

#### Participants

Twenty-five participants (17 women), recruited via the online platform “SONA” at the University of Konstanz, took part in the study in exchange for monetary compensation. The participants were, on average, 21.5 years old, with an age range between 18 and 25 years. All participants reported normal or corrected-to-normal vision. The payment was performance-based and could vary between 8 and 16.6 €. On average, participants earned 9.4 €. The study was performed in accordance with the ethical standards of the Declaration of Helsinki (1964) and its later amendments and with the ethics and safety guidelines of the University of Konstanz. Each participant was informed that the experiment could be abandoned at any time without any repercussions. Informed consent was provided by marking a checkbox on the computer screen. Without check marking, the experiment could not be started.

The required sample size was calculated in G^∗^Power (Faul et al., 2007) for the difference in the Simon effect between the two value-associated conditions (i.e., reward and loss). As there are no prior reports on corresponding effect sizes, we assumed a medium effect size of *d*_*z*_ = 0.5 (Cohen, 1988). For *α =* 0.05 and 1 - *β* = 0.80 (one-tailed), the resulting sample size was 27. Because of some testing restrictions, we ended up with a final sample size of 25 participants.

#### Apparatus

Stimuli were presented on a 23.8-inch color monitor (Fujitsu B24-8TE Pro) with a refresh rate of 60 Hz and a resolution of 1,920 × 1,080 pixels. The distance between participant and monitor was approximately 60 cm. The experiment was programmed in JavaScript, HTML, and CSS. The program ran in Google Chrome (version: 70) on a Windows 10 PC. Participants entered their responses on a German QWERTZ-keyboard (“Y” and “M” key). Each participant was tested together with up to five other participants in a group laboratory.

#### Stimuli and Conditions

Each search display consisted of a target and a distractor, located 2.62° to the left and to the right of the center, respectively. Both items were circles, 1.84° in diameter, enclosing a centered white line (length: 1.31°, width: 0.16°). In the target, the line was oriented either vertically or horizontally, whereas the orientation in the distractor was tilted 45° to the left or right. Target and distractor orientation were randomized. The contour of the target circle was gray (RGB: 149, 149, 149), whereas the contour of the distractor circle could be gray (RGB: 149, 149, 149), blue (RGB: 78, 175, 204), purple (RGB: 180, 130, 204), or brown (RGB: 204, 134, 78). The stimuli were presented on a black background.

We implemented four distractor conditions: gray, no value, reward, and loss. A gray distractor had the same gray color as the target, but was not associated with possible gains or losses. A no value distractor was a color singleton, which signaled that participants could receive zero Eurocents of zero possible cents irrespective of whether their response was correct or within the time limit (see below). Reward and loss distractors were also color singletons. In the reward condition, participants could win 8 Eurocents, if they responded correctly within the time limit. In case of an error or a response after the deadline, they earned nothing. Finally, in the loss condition, participants lost 8 Eurocents in case of a timeout or performance error, but did not lose money, if correct and in time. The mapping between color and the no value, reward, and loss condition was counterbalanced via a Latin square across participants. Each participant started at a base payment of 5 €. A minimum amount of 8 € was paid to each participant at the end of the experiment, of which the participants were unaware before and during the experiment.

#### Procedure

Each trial started with the presentation of a fixation cross (1.31° visual angle) for 400 ms. After a blank screen of 100 ms, the stimuli occurred and remained on the screen until response. The task was to indicate the orientation (vertical or horizontal) of the line in the target by pressing a corresponding key (“Y” or “M”) on the keyboard. The stimulus–response mapping was counterbalanced across participants. Crucially, the participants were required to respond before a prespecified deadline (see below). Errors were signaled by a 100-ms beep. At the end of each trial, visual feedback was given for 1,500 ms, followed by a blank screen for another 500 ms. In the gray condition, only the response time (RT) was provided as feedback. In the other conditions, feedback consisted of the participant’s RT and of the actual (in white) and possible (in the respective color) reward or loss in the current trial (Figure 1).

The experiment began with a practice block of 32 trials with no deadline and where participants could not earn or lose money. Accordingly, only a visual feedback about the RT in the current trial and the acoustic feedback in case of an error were provided. Before the practice block, participants were informed that they should look for the gray circle with a horizontally or vertically oriented line within and categorize this line as horizontal or vertical. After the practice block, they were additionally informed about the different colors of the distractor circle and how they are related to the possible gains and losses, but also that they were irrelevant to the task itself. Furthermore, they were also told which deadline (see below) they had to meet in the following block. Then 26 experimental blocks followed with 32 trials each. Between blocks, participants could take a short rest. After each block, participants were informed about their averaged RT, their error rate, their total earning so far, and updated deadline information. The deadline was adaptive and corresponded to the 75th percentile of the individual RTs in the previous block. The deadline of the first block corresponded to the 75th percentile in the practice block. Only correct responses were used for deadline calculation.

### Results

*A priori*, we set to exclude participants whose error rates were higher than 25% in at least two of the four value conditions in the congruent trials and additionally higher than 50% in at least two of the four value conditions in the incongruent trials. None of the participants were excluded based on these criteria. All analyses were performed with R (R Core Team, 2019). Only responses faster than the deadline (75.3% of all trials) were included in the analyses as we could not rule out that participants used trials in which the deadline was exceeded to rest. Assuming such strategic responding appears plausible based on the data of Kiss et al. (2009), who reported a corresponding behavior in a similar task. In RT analyses, only correct trials were included. We report Greenhouse–Geisser-corrected degrees of freedom (Greenhouse and Geisser, 1959) in case the assumption of sphericity was violated. The mean error rate was 18.3%, and mean RT was 406.1 ms.

#### Response Times

We conducted a repeated-measures analysis of variance (ANOVA) with the factors congruency (congruent vs. incongruent) and condition (gray vs. no value vs. loss vs. reward) to get an overview of the data. The analysis revealed a significant main effect of congruency, *F*(1,24) = 37.39, *p* < 0.001, $\eta_{\text{p}}^{2}$ = 0.61, but the main effect of condition was not significant, *F*(1.34,32.10) = 0.33, *p* = 0.63, $\eta_{\text{p}}^{2}$ = 0.01. The interaction of congruency and condition was very close to significance, *F*(3,72) = 2.70, *p* = 0.052, $\eta_{\text{p}}^{2}$ = 0.10.

**FIGURE 2:**
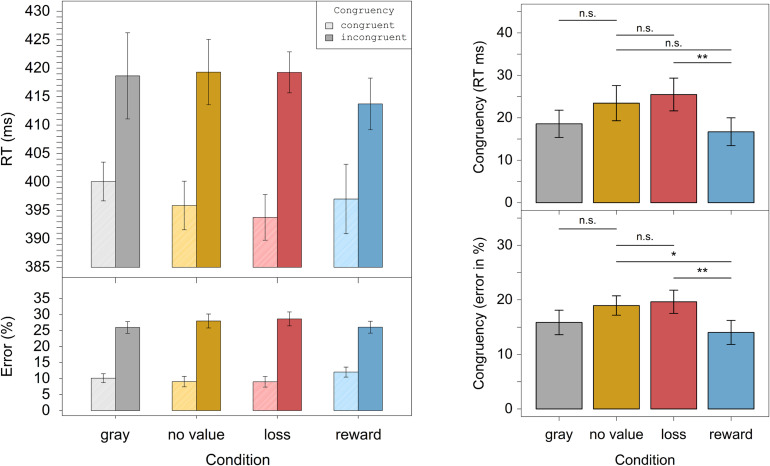
Experiment 1. The left panel displays the RTs and error rates for the different congruency and distractor conditions. The right panel displays differences in the Simon congruency effects for respective pairs of distractor conditions. Displayed are only data from trials in which the deadline was met. Error bars correspond to the within-subject confidence intervals (Morey, 2008). *Significant on *p* < 0.05, **significant on *p* < 0.01, ***significant on *p* < 0.001, n.s. = not significant.

We investigated this interaction in more detail. In a first step, we calculated the Simon effect, i.e., the difference between incongruent and congruent, for each level of the factor condition and each participant separately. To investigate our directional hypotheses concerning the influences of color, reward, and loss association on the Simon effect, we conducted one-tailed planned paired *t*-tests between specific conditions for the Simon effect (cf. Cho and Abe, 2013). The color hypothesis was examined by comparing the gray and no value conditions, which, however, did not differ significantly in the predicted direction, *t*(24) = -1.54, *p* = 0.93, *d*_*z*_ = 0.31. In a next step, we examined possible effects of value by comparing the no value condition with the loss or reward conditions. These comparisons were not significant, *t*(24) = -0.5, *p* = 0.69, *d*_*z*_ = 0.10; and *t*(24) = 1.69, *p* = 0.052, *d*_*z*_ = 0.34, respectively. The Simon effects of the loss and reward conditions differed significantly, *t*(24) = 2.95, *p* = 0.003, *d*_*z*_ = 0.59, which reflected a smaller Simon effect in the reward relative to the loss condition (Figure 2).

#### Error Rates

As for the RTs, we conducted a repeated-measures ANOVA with the factors congruency (congruent vs. incongruent) and condition (gray vs. no value vs. loss vs. reward). The main effect of congruency was significant, *F*(1,24) = 112.86, *p* < 0.001, $\eta_{\text{p}}^{2}$ = 0.82, but the main effect of condition was not, *F*(3,72) = 0.46, *p* = 0.71, $\eta_{\text{p}}^{2}$ = 0.02. More importantly, the analysis revealed a significant interaction of congruency with condition, *F*(3,72) = 3.37, *p* = 0.023, $\eta_{\text{p}}^{2}$ = 0.12.

To disentangle the interaction, we conducted the same comparisons as for the RTs. These analyses revealed no significant difference between the gray and no value condition in the predicted direction, *t*(24) = -1.85, *p* = 0.96, *d*_*z*_ = 0.37. While the Simon effects between the no value and the loss condition did not differ in the predicted direction, *t*(24) = -0.38, *p* = 0.65, *d*_*z*_ = 0.075, there was a significant difference between the no value and reward condition, *t*(24) = 2.35, *p* = 0.014, *d*_*z*_ = 0.47. As can be seen in Figure 2, the Simon effect was reduced in the reward condition relative to the no value condition. As in the RTs, the loss and reward condition also differed significantly, *t*(24) = 2.94, *p* = 0.004, *d*_*z*_ = 0.59, which reflected again a smaller Simon effect in the reward relative to the loss condition (Figure 2).

### Discussion

The aim of Experiment 1 was to examine the effects of value associations on attention and subsequent response selection. As expected, the Simon effect was attenuated in the error rates for reward relative to no value distractors. Moreover, the Simon effect in the reward condition was also reduced compared to the one in the loss condition. Both results indicate that the reward association of distractors influenced attention and subsequent response selection. However, neither did colored distractors reduce the Simon effect relative to gray ones, nor was there a reduction due to loss-associated distractors.

**FIGURE 3:**
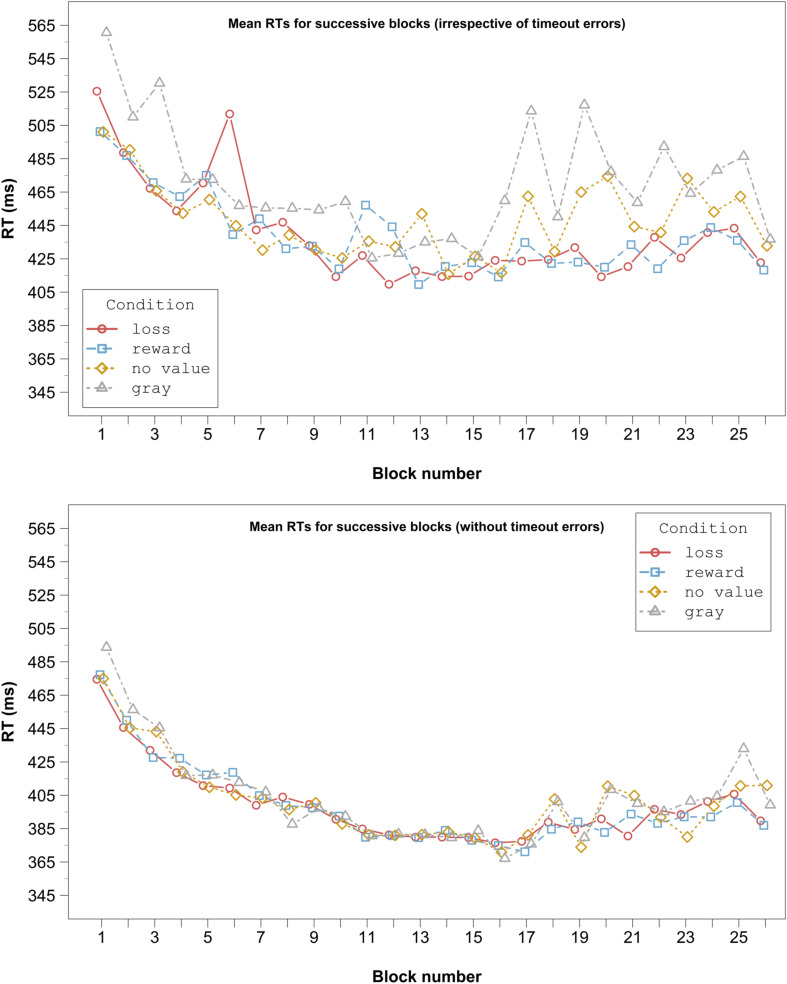
RTs averaged across congruent and incongruent conditions for successive blocks in Experiment 1. The graph in the upper panel shows the complete data, irrespective of timeout errors. The graph in the lower panel displays only data in which the deadline was met. For clarity, error bars are not included.

Because of these unexpected results, we inspected our data visually in a blockwise manner. We did this for the data where participants met the deadline, but also for the whole dataset, as possible interferences might be more obvious in the latter. If considering all data, RTs increased tremendously in the second half of the experiment in the gray and no value conditions (Figure 3, upper panel). This pattern was also still visible in the data in which the deadline was met (Figure 3, lower panel). In the reward and loss conditions, however, this pattern did only emerge slightly (Figure 3, both panels). As only in the latter the receipt of reward or loss was possible, the pattern could hint at participants resting in the gray and no value conditions to adapt their deadline strategically.

Moreover, the visual inspection of the whole dataset revealed that participants seem to respond generally slower in the gray than in the other conditions (Figure 3, upper panel). These longer RTs could be an indicator that an additional process may have been involved (see Proctor and Lu, 1994, for a similar idea). In the gray condition, targets and distractors were gray and thus only distinguishable by the differently tilted lines. A reasonable account is that in order to decide whether the stimulus attended first is the target or the distractor, line orientation had to be categorized. If the distractor was colored, such an additional decision stage was presumably not necessary, because the color immediately signaled that this item is a distractor.

Because of the reasoning above, we conducted a second experiment in which we (a) avoided the supposed additional process in the gray condition and (b) discouraged any attempt to strategically delay responding in the gray and the no value conditions.

## Experiment 2

Experiment 2 was similar to Experiment 1, except that the deadline was adapted only during the first six blocks. Starting with Block 7, the deadline remained constant. This modification should prevent strategic slowing because there was no incentive to manipulate the deadline by doing so after Block 6. Additionally, we removed the line from the distractors, so that it can be decided faster whether a gray item is a target or a distractor. However, a side effect of this modification might be that attention is more often captured directly by the target, because the target is characterized by a unique white line, which then reduces the possibility of finding modulations of the Simon effect by the distractors.

### Methods

#### Participants

To compensate for the possible reduction in absolute size of the Simon effect due to the more frequent direct attentional capture of the target, we increased the necessary sample size to 36 participants. Because two participants had to be excluded because of poor performance (see exclusion criteria of Experiment 1), we recruited 38 participants in the same way as in Experiment 1. The final sample consisted of 30 female and 6 male participants with an average age of 23.6 years (range = 18–29 years). All participants reported normal or corrected-to-normal vision. Participants could earn 8 to 16.6 €, depending on their performance. On average, they earned 10.7 €.

#### Apparatus, Stimuli, and Procedure

Apparatus, stimuli, and procedure were similar as in Experiment 1, except for the following: First, the individual deadlines were only adapted during the first six blocks. Thus, after Block 6, participants were informed about the current deadline and that this deadline would not change anymore. Second, we omitted the line in the distractor circle. Moreover, in the written on-screen instruction, we used an additional equivalent two terms for “horizontal” and “vertical” (i.e., “waagerecht” and “senkrecht”), as some participants in Experiment 1 had difficulties in differentiating the two. We also integrated an additional picture in the instruction depicting the two orientations.

### Results

**FIGURE 4:**
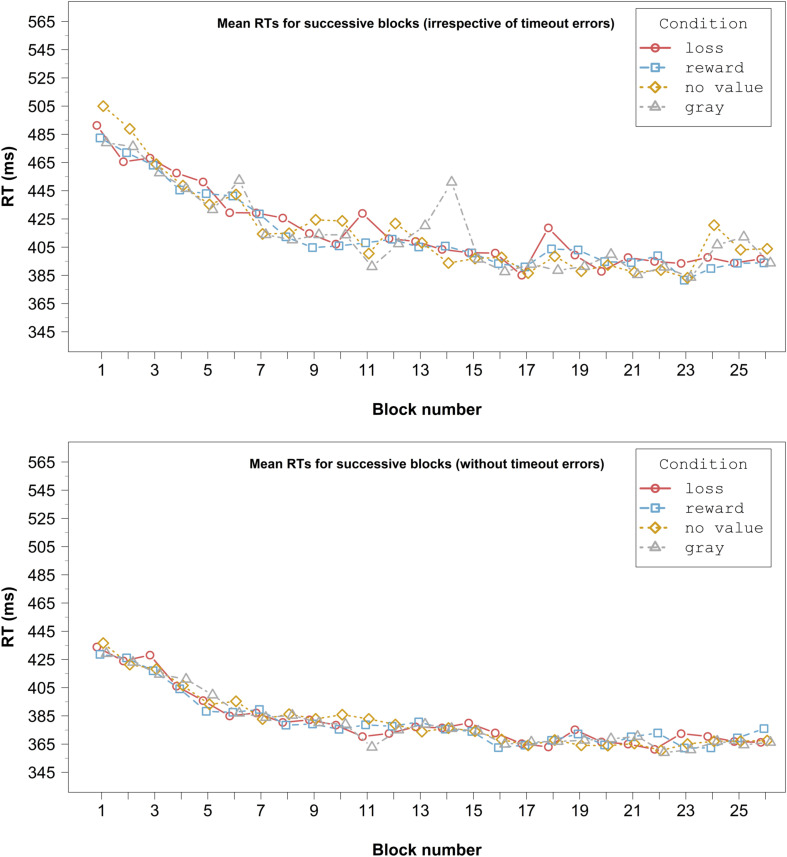
RTs averaged across congruent and incongruent conditions for successive blocks in Experiment 2. The graph in the upper panel shows the complete data, irrespective of timeout errors. The graph in the lower panel displays only data, where the deadline was met. For clarity, error bars are not included.

Only responses faster than the deadline (81.1% of the trials) were analyzed in the same way as in Experiment 1. The mean error rate and RT in the remaining trials were 18.7% and 388.1 ms, respectively. Visual inspection of Figure 4 shows that there was no response slowing in the gray and no value conditions. Moreover, responses in the gray condition were not generally slower than in the other conditions.

#### Response Times

We conducted a repeated-measures ANOVA with the factors congruency and condition. The analysis revealed a significant main effect of congruency, *F*(1,35) = 50.53, *p* < 0.001, $\eta_{\text{p}}^{2}$ = 0.59, as well as a significant interaction of congruency and condition, *F*(3,105) = 5.23, *p* = 0.002, $\eta_{\text{p}}^{2}$ = 0.13. The main effect of condition was not significant, *F*(3,105) = 0.28, *p* = 0.84, $\eta_{\text{p}}^{2}$ < 0.01.

**FIGURE 5:**
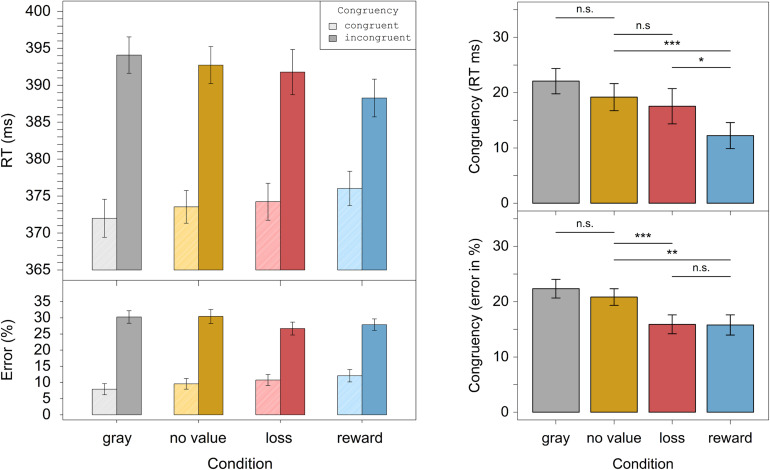
Experiment 2. The left panel displays the RTs and error rates for the different congruency and distractor conditions. The right panel displays differences in the Simon congruency effects for respective pairs of distractor conditions. Displayed are only data from trials in which the deadline was met. Error bars correspond to the within-subject confidence intervals (Morey, 2008). *Significant on *p* < 0.05, **significant on *p* < 0.01, ***significant on *p* < 0.001, n.s. = not significant.

We examined the interaction and tested our directional hypotheses by conducting one-tailed planned paired *t*-tests between the specific conditions. Concerning the effect of color, Figure 5 shows that the Simon effect was numerically increased in the gray condition relative to the no value condition. However, this difference was not significant, *t*(35) = 1.27, *p* = 0.11, *d*_*z*_ = 0.21. Moreover, the Simon effect in the no value condition differed significantly from the one in the reward condition in the predicted direction, *t*(35) = 3.48, *p* < 0.001, *d*_*z*_ = 0.58. In contrast, the Simon effects in the no value condition and the loss condition did not differ significantly in the predicted direction, *t*(35) = 0.54, *p* = 0.30, *d*_*z*_ = 0.09. Again, there was a significant difference between the Simon effect in the loss condition and the one in the reward condition, *t*(35) = 1.82, *p* = 0.039, *d*_*z*_ = 0.30. As can be seen in Figure 5, the Simon effect was attenuated in the reward condition relative to the no value and the loss condition.

#### Error Rates

First, a congruency by condition repeated-measures ANOVA was conducted to get an overview of the data. Besides a significant main effect of congruency, *F*(1,35) = 85.59, *p* < 0.001, $\eta_{\text{p}}^{2}$ = 0.71, there was also a significant congruency by condition interaction, *F*(3,105) = 8.30, *p* < 0.001, $\eta_{\text{p}}^{2}$ = 0.19. The main effect of condition was not significant, *F*(3,105) = 1.57, *p* = 0.20, $\eta_{\text{p}}^{2}$ = 0.04.

The interaction was further investigated using one-tailed planned paired *t*-tests. The analyses revealed that the gray and no value condition did not differ significantly in the predicted direction, *t*(35) = 1.06, *p* = 0.15, *d*_*z*_ = 0.18. The Simon effect in the no value condition differed significantly from the Simon effect in the loss condition, *t*(35) = 3.41, *p* < 0.001, *d*_*z*_ = 0.57, and the one in the reward condition, *t*(35) = 2.79, *p* = 0.004, *d*_*z*_ = 0.47. The Simon effects between the loss and reward conditions, however, did not differ in the predicted direction, *t*(35) = 0.06, *p* = 0.47, *d*_*z*_ = 0.01. Inspection of the data (Figure 5) revealed that the Simon effect was attenuated in the two value conditions relative to the no value one.

### Discussion

In Experiment 2, we eliminated the shortcomings of Experiment 1 (i.e., the possibility to delay responding strategically, and the possible additional process in the gray condition). Visual inspection of the block data (Figure 4) shows that, contrary to Experiment 1, RTs did not increase across the blocks in the gray and no value conditions, which speaks against a strategic slowing. Responses in the gray condition were also not generally slower than in the other conditions.

Although colored distractors numerically reduced the Simon effect relative to gray ones in the RT and the error data, this difference was not significant. Thus, we were not able to conceptually replicate the pattern observed by Proctor and Lu (1994) for colored distractors. We will come to this point again in the *General Discussion*. However, if the colored distractor was additionally associated with reward or loss, the Simon effect was reduced relative to the no value condition. Thus, the value association of a distractor not only seems to boost attentional capture, but also influences response selection. This is in line with studies in which similar effects on selective attention have been found for reward associations (e.g., Anderson et al., 2011a). As in Experiment 1, a reward distractor reduced the Simon effect to a greater extent than a loss distractor in the RTs. This indicates that differences in the value association of the distractors are reflected in a different modulation of response selection.

## General Discussion

In two experiments, we investigated the effects of value associations on attention and response selection. To this aim, we used a modified Simon task, in which visual distraction was supposed to not only affect the search process, but also subsequent response selection, indicated by an attenuation of the Simon effect. We predicted that the more attention is attracted by a distractor, the more the Simon effect should be reduced. Four conditions were realized by corresponding distractor types. One distractor type was gray (same gray as the target), whereas the others were colored. The specific color of the distractor indicated the possible receipt of no value, reward, or loss, respectively.

In Experiment 1, a reward-associated distractor attenuated the Simon effect relative to a colored distractor not associated with any value in the error rates. Moreover, a reward-associated distractor also reduced the Simon effect relative to a loss-associated one in the RTs and the error rates. But no attenuation of the Simon effect due to color or a loss association could be observed. However, inspection of the RTs revealed that in the second half of the experiment, RTs increased, especially in the gray and no value conditions. This suggested that participants deliberately slowed their responses to increase the adaptive deadline, rendering analyses including these two conditions difficult to interpret.

In Experiment 2, to prevent strategic response slowing in later blocks, starting with Block 7 the deadline remained constant. We also removed the tilted line from the distractors, in order to make the target unique in at least one feature (presence of an additional line) also in the gray distractor condition. In Experiment 1, this condition may have required an additional decision stage in processing relative to the other distractor conditions. We assumed that this modification should facilitate the discrimination between target and distractor in Experiment 2.

In Experiment 2, colored distractors numerically reduced the Simon effect relative to the gray distractor, but not significantly so. Thus, we were not able to conceptually replicate the results of Proctor and Lu (1994). However, we found that reward (in the RT and the error rates) as well as loss associations (in the error rates) reduced the Simon effect beyond the only numerical reduction due to color. Moreover, as in Experiment 1, a reward-associated distractor attenuated the Simon effect more than a loss-associated one in the RTs, thus replicating the result of Experiment 1.

### Modulation of the Simon Effect

Given our results, how can the modulation of the Simon effect be explained from a more mechanistic view? In our task, target and distractor are presented on opposite sides of the display. If the distractor is colored, or even associated with value, the activity signal for this location on the common activity map should rise (cf. Failing and Theeuwes, 2018), which increases the probability that the distractor captures attention.

The target could be presented, for instance, on the left and the distractor on the right. According to TEC (cf. Hommel et al., 2001; Hommel, 2019), this should result in the activation of different spatial feature codes for the target (left) and the distractor (right). Because of the lateralization of items and response keys, it can further be assumed that the perceptual representation of a stimulus and corresponding response representations overlap (cf. Hommel et al., 2001; Hommel, 2011). Moreover, it is assumed that attention is needed to bind features correctly in a common percept (cf. Treisman and Gelade, 1980; Treisman, 1998). Thus, attending the distractor should result in a stronger binding of its stimulus and response feature codes, giving rise to an increased activation of the spatial response code, which corresponds to the distractor location. This activation is always opposite to the activation of the target, and therefore, it should modulate a Simon effect elicited by the target location. This is exactly what we observed. Distractors with value-associated colors modulated the Simon effect.

### Effect of Color

Colored distractors did not attenuate the Simon effect relative to gray distractors, neither in Experiment 1 nor in Experiment 2, albeit in the latter, a corresponding pattern was numerically observable in the RTs and the error rates. Thus, in both experiments, we were not able to conceptually replicate the findings of Proctor and Lu (1994). This raises the question: How can this difference between both studies be explained^1^ ?

One possibility is that in a set with two items a colored distractor is not very salient at all, as the target and the distractor are both distinct. This is unlike in visual search tasks where, for instance, a colored item could be presented among multiple gray items. In this kind of task, the gray items did not differ from each other, but the colored item differs from all gray items and has therefore a greater probability to attract attention (see, e.g., Wolfe, 1994). But given that Proctor and Lu (1994) also used only two items in their task, this explanation cannot elucidate why the results in both studies differ. In the study of Proctor and Lu (1994, Experiment 3 and 4), participants had to categorize the lateralized target as either being an “H” or an “S,” whereas the letter “Y” could be presented as distractor on the opposite side of the display. In the conditions in which a distractor was present, the distractor could have either the same color or another color as the target^2^. Thus, the color was integrated in the same element, which had to be categorized. In our task, target and distractor were both circles, which could differ in color. Inside the target, a horizontal or vertical line was present, and participants were required to categorize its orientation. In each distractor, there was either a tilted line (Experiment 1) or no line (Experiment 2). Therefore, the color of the target circle was not necessary to solve the task. Moreover, it was not an integral part of the task-relevant feature/stimulus (the line). Consequently, participants might have been able to suppress the colored distractor to a certain degree in our task (cf. Gaspelin and Luck, 2018). Hence, it is possible that the Simon effect is more reduced if the color (or also value association) is an integral part of the task-relevant feature. Clearly, this issue goes beyond the scope of this article and should be examined in future research.

### Effects of Reward and Loss Associations

In Experiment 2, the Simon effect was attenuated if a colored value-associated distractor was present relative to a colored distractor not associated with value. In our task, the values have never been associated with a task-relevant stimulus. Moreover, although the value-associated distractor signaled the possibility to gain a reward or to lose already earned money, actually attending this distractor resulted in a higher probability to not receiving the reward or to losing money. Our result is in line with other studies in which distractors were associated with reward and in which these reward-associated distractors influenced spatial attention, although never task-relevant (e.g., Le Pelley et al., 2015). Moreover, it fits to the results of Wang et al. (2019), who showed that the Simon effect can be modulated by the reward association of the target.

However, our result extends the existing literature in two ways. First, we show that distractors that were repeatedly associated with a possible loss are also able to interfere with spatial attention in a way that is qualitatively similar to reward associations. Second, the task-irrelevant value-associated distractors affected not only selective attention but also subsequent response selection. Note that an influence of the value association on response selection was also found in the error data. This indicates that task-irrelevant value associations not only primed the response corresponding to the location of the distractor, but it also often even resulted in the execution of this response. This is crucial, given the constant flow of advertisements we are exposed to. It means that advertising not only competes with other stimuli for our attention, but might also influence our actions. This could especially be the case, if we act under time pressure (as in the present study).

In both experiments, reward-associated distractors reduced the Simon effect more than loss-associated ones. To our knowledge, there is only one study in which reward and loss associations were varied within a Simon task, but this was within the target. In the study of van Wouwe et al. (2015), participants learned to associate specific rewards and losses with specific colors in a training task. In a subsequent test, these value-associated colors were presented within the target of a Simon task. However, with this manipulation and contrary to our results, the authors did not find differences between reward- and loss-associated targets in the mean RTs and mean accuracy data. We can only speculate about the origin of the different results. One possible explanation could be the study design: In the test phase of van Wouwe et al. (2015), participants did not earn or lose any money, if the value-associated color was present. In contrast, in our study, participants could win or lose money over the course of the whole experiment. Thus, it is possible that the influence of value associations on response selection might vanish relatively fast, if the value associations are no longer reinforced. It is to future research to investigate this issue.

As mentioned in the *Introduction*, popular performance-contingent manipulations for inducing reward and loss associations do not allow any conclusion, whether the effects emerge due to differences between the reward and loss associations *per se* or due to an imbalance in the frequency of occurrence of the rewards and losses. From our experiments, we can also not draw any conclusions in this respect (and it was also not our aim, see Experiment 1). Thus, it remains for future research to explore whether there are differences between reward and loss from a mechanistic viewpoint. But irrespective of these problems, our results clearly show that differences in the value association of a distractor have an impact not only on attention, but also on subsequent response selection.

## Conclusion

Our results extend already existing literature (e.g., Le Pelley et al., 2015) by showing that monetary loss, which was continuously associated with the occurrence of a specific distractor feature, can also influence spatial attention in a similar manner than monetary reward. But more importantly, our results clearly demonstrate that both stimulus–value associations affect not only selective attention, but also subsequent response selection. Moreover, differences in the value association of stimuli are also reflected in a different impact on response selection.

## Data Availability Statement

The datasets presented in this study can be found in online repositories. The names of the repository/repositories and accession number(s) can be found below: https://osf.io/p5ngj/.

## Ethics Statement

Ethical review and approval was not required for the study on human participants in accordance with the local legislation and institutional requirements. The patients/participants provided their written informed consent to participate in this study.

## Author Contributions

AW: literature review and data analysis. AW, MD, and RH: idea generation, design of behavioral experiments, interpretation of results, preparation of draft manuscript, and preparation of final manuscript. AW and MD: formulating hypotheses. All authors contributed to the article and approved the submitted version.

## Conflict of Interest

The authors declare that the research was conducted in the absence of any commercial or financial relationships that could be construed as a potential conflict of interest.

## Footnotes

1 We thank both reviewers for pointing us to these two explanations.

2 Since in the study of Proctor and Lu (1994) many additional conditions were varied especially with regard to the color or presence of a fixation point, we only refer to the conditions here, where the target and the distractor could be uniquely separated by their color.
